# National Health Insurance unpacked: Part 1

**DOI:** 10.4102/safp.v62i1.5094

**Published:** 2020-02-13

**Authors:** Bob Mash

**Affiliations:** 1 Division of Family Medicine and Primary Care, Faculty of Medicine and Health Sciences, University of Stellenbosch, Cape Town, South Africa

## Introduction

This new four-part series of the *South African Family Practice* journal will provide some details of the National Health Insurance (NHI) as proposed in the NHI bill that was laid before the Parliament in July 2019.^1^

Research published in local journals has shown that many general practitioners in both urban and rural areas need more accurate information on NHI so that they can understand what it means for them.^2,3^ A survey involving members of the South African Academy of Family Physicians conducted in 2019 also showed that many doctors were misinformed, uninformed and very concerned about what NHI might mean for their practices and their role in the health system.^4^

This series, therefore, intends to provide up-to-date information on NHI and what is really planned. While the academy is supportive of the values and principles behind NHI, we are aware of the widespread concern and scepticism among our members about its actual implementation.

Dr Nicholas Crisp agreed to assist me to help ensure that the contents of the series are accurate and up to date. Dr Crisp is a consultant for the NHI Fund Office at the Ministry of Health in Pretoria, South Africa.

In the first part of this series, we focus on the big picture issues of governance and funding. In the following three parts, we will focus on the issues most immediately relevant to general practitioners, medical officers and family physicians working in primary care. Part 2 will focus on accreditation of primary care facilities, Part 3 will focus on registration of patients and Part 4 will focus on remuneration of primary care providers.

## What is National Health Insurance?

National Health Insurance is essentially a medium for funding healthcare for all the people of South Africa. It will enable universal health coverage in South Africa by ensuring that services are free for everyone at the point of use and are obtained from providers who meet the stated quality criteria.

National Health Insurance intends to correct imbalances, injustices and inequities of the past and to build a society based on social justice and fundamental human rights as described in the Constitution of South Africa.

The NHI funds will be obtained from the national fiscus through taxes, which may include mandatory prepayments. The funds will be used for the strategic purchasing of healthcare services, medicines, health goods and related products from accredited and contracted healthcare service providers. Service providers may be chosen from the current private or public healthcare sectors.

## Who is covered by National Health Insurance?

All South African citizens, permanent residents, refugees and inmates will be covered by the NHI. All children regardless of origin will be covered. Asylum seekers, illegal migrants and foreigners will only be covered for emergencies and notifiable conditions. Visiting foreigners will be covered by their own travel insurance.

## How will National Health Insurance be funded?

Money for the NHI fund will be collected from the following sources:

- Reallocation of medical scheme tax credits. At present, those who pay contributions to medical schemes are able to lay claims for tax returns. In future, we will not need to claim for returns as NHI will cover us and the money will be reallocated to NHI.
- General tax revenue and shifting funds from what is currently given to the provinces to deliver health services.
- A payroll tax may be introduced and for many people this would replace their current medical aid contributions.
- A surcharge on personal income tax.

## Can we afford National Health Insurance?

South Africa currently ‘affords’ 8.4% of its gross domestic product (GDP) on healthcare. The return on investment is poor by global standards, so affordability is relative. We should be using this money more efficiently and effectively. The fund will have massive buying power, thus securing better prices based on volume. Management of the change to NHI will require additional investment. How much is required will depend on the pace of change and the interventions chosen. By 2026 it is estimated that the change to NHI will cost R33 billion.

## How will National Health Insurance be governed?

Governance and stewardship of the fund as well as the health system are ultimately the responsibility of the Minister of Health.

The NHI scheme will be accountable to a board comprising 11 members who would be appointed by the Minister for a 5-year period. The Minister must select people only from a list of candidates nominated by the public. Candidates must be publicly interviewed and recommendations must be made by an advisory panel that is selected by the Minister. The Board may then establish a number of technical committees. The NHI fund will have a Chief Executive Officer who is accountable to the Board for the administration of the fund.

The minister will also establish a number of other advisory committees:

- Benefits Advisory Committee: this committee will advise on the benefits covered by NHI. This would include expertise in medicine, public health, health economics, epidemiology and patients’ rights.
- Health Care Benefits Pricing Committee: this committee will advise on what to pay providers for the various benefits. This would include expertise in actuarial science, medicines, epidemiology, health management, health economics, health financing, labour and patients’ rights.
- Stakeholder Advisory Committee: this committee would provide advice on NHI with the help of key stakeholders, such as health professions councils, health public entities, organised labour, civil society, associations of health professionals and providers as well as patient advocacy groups.

## What then is the role of national, provincial and district health structures?

The National Department of Health will be responsible for the healthcare policy and strategy in the country. This will include publishing guidelines, planning human resources for health, ensuring integration of a comprehensive health system and planning new infrastructure.

The provincial departments’ role will change as the NHI fund will be directly accessed by providers (hospitals and sub-districts) for health services. Provinces will have an oversight role and will still be responsible for public ambulance services.

The district management teams will focus on the quality of health services in their area.

## What then is the role of medical aid schemes?

In future, once NHI is fully implemented, additional insurance through medical schemes will be limited to services not already covered by NHI.

## How will National Health Insurance be implemented?

National Health Insurance will be phased gradually, using a progressive and programmatic approach, based on financial resource availability. The current phase involves system strengthening for the next 5–6 years. This means that there will be investment in quality of care, infrastructure, information systems as well as administrative and operational processes in both the public and private sectors. Key aspects of the NHI system will be field-tested, consulted on and published.
